# White Matter Structural Network Analysis to Differentiate Alzheimer’s Disease and Subcortical Ischemic Vascular Dementia

**DOI:** 10.3389/fnagi.2021.650377

**Published:** 2021-03-31

**Authors:** Mengmeng Feng, Yue Zhang, Yuanqing Liu, Zhiwei Wu, Ziyang Song, Mengya Ma, Yueju Wang, Hui Dai

**Affiliations:** ^1^Department of Radiology, The First Affiliated Hospital of Soochow University, Suzhou City, China; ^2^Department of Geratology, The First Affiliated Hospital of Soochow University, Suzhou City, China; ^3^Institute of Medical Imaging, Soochow University, Suzhou City, China

**Keywords:** Alzheimer’s disease, subcortical ischemic vascular dementia, diffusion tensor imaging, structural network analysis, graph theory method

## Abstract

To explore the evaluation of white matter structural network analysis in the differentiation of Alzheimer’s disease (AD) and subcortical ischemic vascular dementia (SIVD), 67 participants [31 AD patients, 19 SIVD patients, and 19 normal control (NC)] were enrolled in this study. Each participant underwent 3.0T MRI scanning. Diffusion tensor imaging (DTI) data were analyzed by graph theory (GRETNA toolbox). Statistical analyses of global parameters [gamma, sigma, lambda, global shortest path length (Lp), global efficiency (E_g_), and local efficiency (E_loc_)] and nodal parameters [betweenness centrality (BC)] were obtained. Network-based statistical analysis (NBS) was employed to analyze the group differences of structural connections. The diagnosis efficiency of nodal BC in identifying different types of dementia was assessed by receiver operating characteristic (ROC) analysis. There were no significant differences of gender and years of education among the groups. There were no significant differences of sigma and gamma in AD vs. NC and SIVD vs. NC, whereas the E_g_ values of AD and SIVD were statistically decreased, and the lambda values were increased. The BC of the frontal cortex, left superior parietal gyrus, and left precuneus in AD patients were obviously reduced, while the BC of the prefrontal and subcortical regions were decreased in SIVD patients, compared with NC. SIVD patients had decreased structural connections in the frontal, prefrontal, and subcortical regions, while AD patients had decreased structural connections in the temporal and occipital regions and increased structural connections in the frontal and prefrontal regions. The highest area under curve (AUC) of BC was 0.946 in the right putamen for AD vs. SIVD. White matter structural network analysis may be a potential and promising method, and the topological changes of the network, especially the BC change in the right putamen, were valuable in differentiating AD and SIVD patients.

## Introduction

According to a report from the World Health Organization in 2017, almost 50 million people have been diagnosed with dementia, and the number is said to increase to 82 million by 2030 (The Lancet Neurology, 2018). Dementia creates a heavy financial burden on society. Alzheimer’s disease (AD) is a progressive and degenerative disease resulting in cognitive impairment and behavior dysfunction. Vascular dementia (VaD) due to various vascular pathologies is the second most common cause of dementia after AD (Kang et al., 2016). AD and VaD accounts for approximately 60% and 20% of dementia, respectively (Rizzi et al., 2014). Subcortical ischemic vascular dementia (SIVD) accounts for a large part of VaD (Benjamin et al., 2016). SIVD has been focused on due to its high prevalence.

It is hard to identify SIVD and AD clinically, due to the similar neuropsychological symptoms between them. The main issues for SIVD patients are executive and semantic memory dysfunction (Palesi et al., 2018). Previous studies demonstrated that the cognitive impairment of SIVD was related to the disconnection of the frontal subcortical circuit (Seo et al., 2010). The progression of SIVD was reversible as the vascular risk factors of SIVD were controllable. However, AD patients mostly displayed diffuse cortex atrophy and the progress was irreversible (McDonald et al., 2009).

The microstructure of the human brain has been explored *in vivo* by neuroimaging in recent years. Many studies have proved that the cognitive impairment of SIVD resulted from a lesion in the white matter (Chen et al., 2018). Diffusion tensor imaging (DTI) was acknowledged as a precise MRI method, which is sensitive to the microstructural change of white matter. In the past couple of decades, the topological network has received great attention in neuroscience study. The network is comprised of information of relations or interconnections that link many elements in the neurobiological system. The neurons of the human brain are interconnected through synaptic connections and anatomical projections mediate the communication among brain areas, forming a highly complex network system in human beings (Bassett and Sporns, 2017; Kuang et al., 2020). Structural network analysis based on DTI is a newly-developed method to reflect the topological structural alterations and the connectivity of the brain structural network (Bassett et al., 2017). The graph theory method, a branch of mathematics, could offer important new insights into the structure and function of brain network systems including their architecture, evolution, and development (Sporns, 2018).

DTI has been applied in many studies to explore white matter changes in patients with AD and SIVD by voxel- or tract-based methods (Chen et al., 2018; Palesi et al., 2018; Liu et al., 2019). However, the human brain is a complicated and interconnected network balancing regional segregation and the specialization of function with strong integration (Zhou et al., 2020). The clinical syndromes of AD and SIVD, such as executive dysfunction and memory deficits, might result from abnormal structural connections. Due to the analogical clinical manifestation of AD and SIVD, it is hard to identify them by only relying on clinical performances. Brain network analysis may help researchers understand cerebral microstructure change, and may further elucidate the etiology of cognitive and behavioral deficits in SIVD patients. In the present study, we aim to explore brain structural network alterations in AD and SIVD patients, and to explore the value of brain structural network analysis in the differentiation of AD and SIVD.

## Materials and Methods

### Participants

A total of 67 right-handed Chinese Han subjects [19 SIVD, 31 AD, and 17 normal control (NC)] were enrolled in this study at the Department of Radiology in the First Affiliated Hospital of Soochow University from June 2018 to June 2020. This study was approved by the ethics committees of the First Affiliated Hospital of Soochow University, and written informed consent was obtained from each subject prior to participation. All subjects underwent a comprehensive neuropsychological test and 3.0 Tesla MRI scanning of the whole brain. The cognitive functions of all the subjects were evaluated by an experienced neuropsychologist. General cognitive function of participants was evaluated using the Beijing version of the Montreal cognitive assessment (MoCA) and the mini mental state examination (MMSE; Lu et al., 2011). Episodic memory function was assessed by the auditory verbal learning test Huashan version (AVLT) including the auditory verbal learning test immediate recall (AVLT-IR) and the auditory verbal learning test delayed recall (AVLT-DR; Zhao et al., 2012). Executive function was assessed by the Stroop color-word test. The speed (Stroop test 1) and the accuracy (Stroop test 2) of performance were measured.

Included SIVD patients met the following criteria (Román et al., 2002; Liu et al., 2014; Kang et al., 2016): (1) displayed moderate and severe white matter hyperintensities (WMH). The severity of WMH was evaluated according to the modified Fazekas ischemia criteria. WMH were assessed separately as periventricular white matter hyperintensities (PWMH) and deep white matter hyperintensities (DWMH) in T2 axial or fluid-attenuated inversion recovery (FLAIR) images. DWMH were classified into D1 (<10 mm), D2 (≥10 mm <25 mm), or D3 (≥25 mm) based on the longest diameter of lesions. PWMH were split into P1 (cap and *band* < 5 mm), P2 (≥5 mm, <10 mm), or P3 (cap or band ≥10 mm) based on the maximum length, which were perpendicular and horizontal to the ventricle, respectively. These two ratings were combined as minimal (D1P1, D1P2), moderate (D1P3, D2P1, D2P2, D2P3, D3P1, D3P2), or severe (D3P3); (2) lacunar cases: multiple lacunas (>5) in the deep gray matter and at least moderate white matter lesions; and (3) with a MoCA score less than 26.

The inclusion criteria for AD patients referred to the National Institute on Aging-Alzheimer’s Association Criteria (McKhann et al., 2011). Besides, AD patients in our study also met following criteria: (1) absence of WMH or mild severity of WMH by T2 FLAIR image; and (2) with a MoCA score less than 26.

According to the score of MoCA, the AD patients and SIVD patients were subdivided into mild cognitive impairment (18≤MoCA< 26) and dementia (MoCA≤17).

NC were age and gender-matched healthy volunteers: (1) without clinical evidence or history of cognitive disfunction with MoCA≥26; (2) without brain abnormality detected on a routine non-contrast MRI examination; and (3) no neuropsychological disorders.

The exclusion criteria for all participants were as follows: (1) metabolic conditions, such as hypothyroidism or folic acid deficiencies; (2) a history of stroke; (3) central nervous system diseases that could cause cognitive decline, such as Parkinson’s disease, epilepsy, multiple sclerosis and so on; and (4) with MRI scanning contraindications.

### Image Acquisition

All MRI examinations were performed using a 3.0 T MRI scanner (Signa HDxt, GE Healthcare, Milwaukee, WI, USA) with an eight-channel head coil. A three-dimensional fast spoiled gradient recalled (3D-FSPGR) sequence was performed with the following parameters: repetition time (TR) 6.50 ms, echo time (TE) 2.80 ms, inversion time (TI) 900 ms, flip angle 8°, field of view (FOV) 256 × 256 mm, number of slices 176, slice thickness 1 mm without slice gap, and scan time 4 min. DTI data were obtained using an echo planar imaging (EPI) sequence with the following parameters: TR 17,000 ms, TE 85.4 ms, flip angle 90°, matrix size 128 × 128, FOV 256 × 256 mm, slice thickness 2 mm without slice gap, number of signal averages (NEX) 2, with 30 non-collinear directions of diffusion encoding (*b* = 1,000 s/mm^2^ for each direction), and a scan time of 9 min. Additionally, axial T2-weighted and FLAIR sequences were obtained to detect visible white matter damage.

### Data Processing

The PANDA toolbox based on FMRIB Software Library v5.0 was applied in tha DTI data process (Cui et al., 2013), containing several steps (brain extraction, DTI images format conversion, realignment, eddy current and motion artifact correction, fractional anisotropy (FA) calculation, and diffusion tensor tractography). When tracking white matter fibers, a fractional anisotropy (FA) value threshold of 0.2 and a turning angle threshold of 45° of the Fiber Assignment by Continuous Tracking (FACT) algorithm were set (Basser et al., 2000).

The Anatomical Automatic Labeling (AAL) atlas was used to parcellate each brain into 90 regions of interest (ROIs). The nodes of the structural network were defined according to the AAL template. Interconnections between brain regions were taken as the edges of the structural network. If the number of interconnected white matter fibers was more than 3, an edge in the structural network was defined. The global topological parameters including the small-world [gamma, sigma, lambda, and global shortest path length (Lp)], global efficiency (E_g_), and local efficiency (E_loc_) were obtained by the GRETNA toolbox (Wang et al., 2015). E_g_ indicates how efficiency information is exchanged over the whole network. E_loc_ calculates clustering and specialization within a network and the fault tolerance of the network. Lp, a measurement of the average nodal shortest path length, reflects the speed information transfer to the whole brain. Sigma, the ratio of gamma and lambda, is a measurement of the small-world property of the network. In addition, the betweenness centrality (BC) of a node was measured to describe nodal characteristics of the white matter structural network. BC is the fraction of all shortest paths in the network that pass through a given node.

### Statistical Analysis

#### Demographics and Clinical Variables

All statistical analyses were performed using the Statistical Product and Service Software (SPSS ver. 20.0, Chicago, IL, USA). The normality of distribution of continuous variables was examined by the Kolmogorov–Smirnov test. The differences of age and years of education among groups were analyzed by the Kruskal–Wallis H test. The differences of MMSE, MoCA, AVLT-IR, AVLT-DR, and Stroop tests 1 and 2 among groups were analyzed by general linear modeling, with some effects for covariates (age, gender, and years of education); and Bonferroni correction was used to adjust *p* values in multiple comparisons. The distribution of cognitive severity between AD patients and SIVD patients were analyzed by the *χ*^2^ test. The group differences of categorical variables were analyzed by the Pearson test when the sample size was over 40 and the minimal expected frequency was over 5. Otherwise, the correction formula of chi-squared test would be chosen; and the R×C table was used when the dependent variable was over 2. A *p* value less than 0.05 was considered statistically significant and continuous variables were reported as mean ±SD.

#### Comparison of Topographic Network Parameters Among Groups

Statistical analyses of global and nodal parameters were performed with the gretna toolbox. Between the two groups, global network parameters and BC of each node were compared by a two-sample *t*-test with FDR correction and with age, gender, and years of education as covariates. For global and nodal parameters, a *p*-value less than 0.001 was considered statistically significant. In order to evaluate the diagnostic accuracy of BC in nodes with group differences in identifying AD patients and SIVD patients, the receiver operating characteristic (ROC) analysis was performed and the area under curve (AUC) was calculated by MedCalc (MedCalc statistical software, ver.15.8).

#### White Matter Structural Connectome Comparison Between Groups

Network-based statistical analysis (NBS), a methodology improving the statistical power though controlling the type I error, was applied to identify the change of structural connections in AD patients vs. NC and SIVD patients vs. NC. Statistical comparisons in NBS were conducted with age, sex, and years of education as covariates. A *p*-value less than 0.05 was considered statistically significant.

## Results

### Demographic and Neuropsychological Characteristics

The demographic information and neuropsychological performance of AD and SIVD patients are shown in Table 1. As shown in Table 1, there were no significant differences of gender and years of education among the three groups. Compared with NC, the scores of MoCA, MMSE, AVLT-IR, and AVLT-DR of AD and SIVD groups were significantly decreased. When AD patients compared with SIVD patients and NC compared with SIVD patients, Stroop tests 1 and 2 of SIVD patients were significantly reduced. However, there were no significant differences in scores of MMSE, MoCA, AVLT-IR, and AVLT-DR in AD patients vs. SIVD patients. There were no significant differences in the scores of Stroop tests 1 and 2 between AD patients and NC. There were respectively 19 AD patients and 14 SIVD patients with dementia and there was no statistical difference in the distribution of severity of cognitive dysfunction between AD and SIVD patients (*p* = 0.369).

**Table 1:** The group difference of clinical and neuropsychological data among AD, SIVD patients, and NC.

Variables	AD	SIVD	NC	*p*-value		
				AD vs. NC	SIVD vs. NC	AD vs. SIVD
Gender (female/male)	17/14	9/10	11/6	NS	NS	NS
Age (years)	72.19 ± 8.90	79.10 ± 7.07	68.06 ± 8.33	NS	<0.001	0.004
Education (years)	9.03 ± 6.11	10.37 ± 5.16	10.53 ± 3.39	NS	NS	NS
MMSE	20.29 ± 6.61	19.05 ± 5.61	27.47 ± 2.98	<0.001	<0.001	NS
MoCA	15.13 ± 6.31	13.89 ± 5.67	25.64 ± 3.37	<0.001	<0.001	NS
AVLT-IR	22.90 ± 11.37	14.26 ± 8.11	42.17 ± 12.14	<0.001	<0.001	NS
AVLT-DR	2.58 ± 3.16	0.368 ± 0.89	8.94 ± 4.31	<0.001	<0.001	NS
Stroop test 1 (s)	35.07 ± 11.23	49.74 ± 29.29	23.48 ± 6.53	NS	<0.001	0.008
Stroop test 2	0.81 ± 1.53	1.84 ± 2.36	0.24 ± 0.66	NS	<0.003	0.01

Note: AD, Alzheimer’s disease; SIVD, subcortical ischemic vascular dementia; NC, normal control; AVLT-IR, the auditory verbal learning test immediate recall; AVLT-DR, the auditory verbal learning test delayed recall; MoCA, Montreal cognitive assessment; MMSE, Mini Mental State Examination; NS, no significance.

### Between-Group Difference in the Global Parameters of the Network

All the subjects performed small-world organization (sigma>1) in the DTI structural network. As shown in Table 2, there were no statistical significances in sigma and gamma when the AD group was compared with NC and the SIVD group was compared with NC. The E_g_ of AD patients and SIVD patients were statistically decreased and their lambda values were increased. The E_loc_ of the SIVD group was significantly reduced in SIVD patients vs. NC. Moreover, the lambda of SIVD patients was also increased when SIVD patients were compared with AD patients. SIVD patients showed significantly increased Lp when the SIVD group was compared with NC and the SIVD group was compared with the AD group. Compared with AD patients, the E_g_and E_loc_ in SIVD patients were reduced.

**Table 2:** The group difference of topological parameters by network analysis.

Graph metrics	AD	SIVD	NC	AD vs. NC	SIVD vs. NC	AD vs. SIVD
E_glob_	0.209 ± 0.021	0.129 ± 0.027	0.243 ± 0.018	<0.001	<0.001	<0.001
E_loc_	0.249 ± 0.050	0.137 ± 0.034	0.294 ± 0.052	NS	<0.001	<0.001
Lp	4.966 ± 0.623	8.190 ± 2.320	4.140 ± 0.323	NS	<0.001	<0.001
Gamma	8.065 ± 1.157	7.710 ± 2.353	7.628 ± 0.894	NS	NS	NS
Lambda	1.223 ± 0.053	1.370 ± 0.199	1.177 ± 0.030	0.001	<0.001	<0.001
Sigma	6.584 ± 0.841	5.610 ± 1.375	6.472 ± 0.654	NS	NS	NS

Note: E_glob_, global efficiency; E_loc_, local efficiency; NS, no significance; AD, Alzheimer’s disease; SIVD, subcortical ischemic vascular dementia; NC, normal control.

### Between-Group Difference in Nodal Parameters

**Figure 1:**
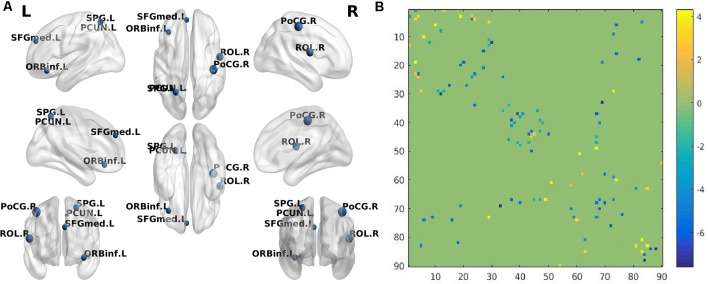
**(A)** The brain regions (nodes) visualized by BrainNet Viewer (NKLCNL, Beijing Normal University) demonstrate significantly different betweenness centrality (BC) when Alzheimer’s disease (AD) was compared with normal control (NC; *p* < 0.001, FDR-corrected). ORBinf.L: left frontal_inf_orb; ROL.R: right rolandic_oper; SFGmed.L: left frontal_sup_medial; PoCG.R: right postcentral; SPG.L: left parietal_sup; PCUN.L: left precuneus. **(B)** The structural connectivity alterations between the AD and NC groups. Horizontal axis and vertical axis respectively represent 90 brain regions in the automated anatomical labeling (AAL) template. The dots in different locations in the matrix represent the structural connectivity between the two different brain regions identified by the horizontal axis and vertical axis. The colors of the dots represent structural connection strength, quantified by the values on the right of the color bar. The dot color with a positive value represents increased structural connection between the two brain regions in the AD group, compared with the NC group, while the dot color with a negative value represents a decreased structural connection.

**Figure 2:**
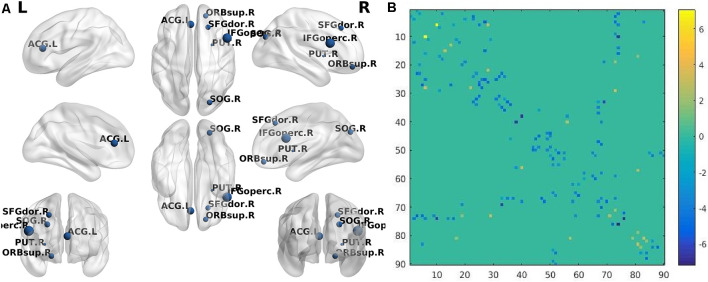
**(A)** The brain regions (nodes) visualized by BrainNet Viewer (NKLCNL, Beijing Normal University) demonstrate significantly different betweenness centrality (BC) when subcortical ischemic vascular dementia (SIVD) was compared with NC (*p* < 0.001, FDR-corrected). SFGdor.R: right frontal_sup; ORBsup.R: right frontal_sup_orb; IFGoperc.R: right frontal_inf_oper; ACG.L: left cingulum_ant; SOG.R: right occipital_sup; PUT.R: right putamen. **(B)** The structural connectivity alterations between the SIVD and NC groups. Horizontal axis and vertical axis respectively represent 90 brain regions in the automated anatomical labeling (AAL) template. The dots in different locations in the matrix represent the structural connectivity between the two different brain regions identified by horizontal axis and vertical axis. The colors of the dots represent structural connection strength, quantified by the values on the right of the color bar. The dot color with a positive value represents increased structural connection between the two brain regions in the SIVD group, compared with the NC group, while the dot color with a negative value represents a decreased structural connection.

When AD patients were compared with NC, the BC of prefrontal cortices such as the left inferior frontal gyrus (orbital part) and left superior frontal gyrus (medial part) in the AD group were decreased significantly. Besides, AD patients had significantly decreased BC in the right rolandic operculum, right postcentral, left superior parietal gyrus, and left precuneus (Table 3; Figure 1A). Compared with NC, SIVD patients demonstrated decreased BC mainly in prefrontal regions, such as the right superior frontal gyrus, right superior frontal gyrus (orbital part), right inferior frontal gyrus (opercular part), and left anterior cingulate gyrus, as well as in the right putamen and right superior occipital gurus (Table 4 and Figure 2A).

**Table 3:** The group difference of nodal betweenness centrality in AD vs. NC and the diagnostic efficiency of the nodes in the differentiation between groups.

Node	AD	NC	AD vs. NC	AUC
Left frontal_inf_orb	37.56 ± 34.42	98.90 ± 48.34	<0.001	0.849
Right rolandic_oper	69.45 ± 52.95	168.91 ± 89.97	<0.001	0.843
Left frontal_sup_medial	124.75 ± 94.75	329.55 ± 230.68	<0.001	0.770
Right postcentral	366.85 ± 186.90	586.96 ± 227.75	<0.001	0.766
Left parietal_sup	213.91 ± 194.41	455.34 ± 226.25	<0.001	0.843
Left precuneus	475.28 ± 162.72	978.91 ± 267.10	<0.001	0.935

Note: AD, Alzheimer’s disease; NC, normal control; AUC, area under curve.

**Table 4:** The group difference of nodal betweenness centrality in SIVD vs. NC and the diagnostic efficiency of the nodes in the differentiation.

Node	SIVD	NC	SIVD vs. NC	AUC
Right frontal_sup	57.73 ± 72.07	371.26 ± 215.27	<0.001	0.937
Right frontal_sup_orb	51.13 ± 51.34	233.86 ± 88.80	<0.001	0.931
Right frontal_inf_oper	11.75 ± 22.65	28.14 ± 19.24	<0.001	0.783
Left cingulum_ant	87.05 ± 78.52	272.13 ± 136.16	<0.001	0.857
Right occipital_sup	37.54 ± 35.08	262.68 ± 137.26	<0.001	0.934
Right putamen	230.00 ± 132.35	605.22 ± 123.79	<0.001	0.9 74

Note: SIVD, subcortical ischemic vascular dementia; NC, normal control; AUC, area under curve.

### Between-Group Difference in Structural Connectivity

Compared with NC, the AD group demonstrated 41 significantly decreased structural connections and 18 increased structural connections. The decreased structural connections of AD patients mainly involved temporal and occipital regions, while approximately 50% of increased structural connections involved frontal and prefrontal regions (Figure 1B). When SIVD patients were compared with NC, SIVD patients manifested 80 significantly decreased structural connections and 13 increased structural connections. Structural connections between frontal and prefrontal regions were significantly decreased in SIVD patients. Additionally, the structural connections between frontal-subcortical regions and prefrontal-subcortical regions were also decreased (Figure 2B).

### Discriminative Power of BC Among AD, SIVD, and NC Groups

The AUC of BC in significantly different cortices between AD patients and NC ranged from 0.766 to 0.935. As shown in Table 3; the AUC of BC in the left precuneus was 0.935 which was the highest. When SIVD was compared with NC, the AUC of BC in right putamen was 0.974 which was the highest (Table 4). As shown in Table 5; the AUC of BC in significantly different nodes (from the SIVD group vs. the NC group and the AD group vs. the NC group) in identifying AD patients and SIVD patients ranged from 0.599 to 0.946; and the AUC of BC in the right putamen was 0.946 which was the highest (Table 5).

**Table 5:** The diagnostic efficiency of nodal betweenness centrality in differentiating SIVD and AD.

Node	AD	SIVD	AUC
Left frontal_inf_orb	37.56 ± 34.42	94.47 ± 57.33	0.744
Right rolandic_oper	69.45 ± 52.95	259.94 ± 197.12	0.864
Left frontal_sup_medial	124.75 ± 94.75	84.71 ± 67.41	0.664
Right postcentral	366.85 ± 186.90	550.69 ± 272.83	0.708
Left parietal_sup	213.91 ± 194.41	347.73 ± 296.25	0.599
Left precuneus	475.28 ± 162.72	641.46 ± 458.63	0.616
Right frontal_sup	598.49 ± 359.91	57.73 ± 72.07	0.940
Right frontal_sup_orb	198.31 ± 156.54	51.13 ± 51.34	0.847
Right frontal_inf_oper	17.29 ± 23.03	11.75 ± 22.65	0.670
Left cingulum_ant	402.22 ± 197.43	87.05 ± 78.52	0.939
Right occipital_sup	363.68 ± 275.23	37.54 ± 35.08	0.933
Right putamen	610.14 ± 222.58	230.00 ± 132.35	0.946

Note: The nodes in this table refer to Tables s 3, 4. AD, Alzheimer’s disease; SIVD, subcortical ischemic vascular dementia; AUC, area under curve.

## Discussion

According to the present study, executive dysfunction was significantly different between the SIVD and AD groups. SIVD patients performed worse in the Stroop color-word tests than AD patients. SIVD patients spent more time on Stroop test 1 (lower speed) and naming/reading errors (lower accuracy) occurred more frequently than in AD patients, as shown in Table 1. It was indicated that the executive function of SIVD patients was more seriously damaged. Subcortical vascular pathology which interrupted frontal-striatal circuits was frequently present in SIVD patients. Executive dysfunction in SIVD patients may result from the disruption of cortical and subcortical connections (O’Brien and Thomas, 2015; Tuladhar et al., 2015). Many studies suggested that memory deficit was more obvious in AD patients. However, the SIVD group performed worse than the AD group in the present study (Graham et al., 2004; Reed et al., 2007). Liu et al. (2019) found that memory loss existed in the deteriorated process of SIVD patients and memory decreased with cognitive deterioration. Moreover, there were studies that proved that memory deterioration was correlated with the overall severity of dementia (Kang et al., 2016). Thus, the discrepancy supposedly resulted from the impartial distribution of global cognitive severity.

DTI and the graph theory method were applied to explore the structural network alteration of AD and SIVD patients. In the present study, AD and SIVD patients and NC performed small-world properties. Small-world organization, reflecting an optimal balance of integration and segregation, appears to be a ubiquitous organization of anatomical connectivity (Bassett and Bullmore, 2006; Zhou et al., 2020). Sigma is a measurement of small-world property in the structural network (Sun et al., 2017). The decreased sigma in the structural network indicates a less optimal topological structure. Although there were no significant differences in sigma between AD and NC and SIVD and NC, it did not indicate that the small-world topological properties of AD and SIVD were normal. Because sigma was calculated by dividing gamma by lambda, the sigma value could be affected both by the gamma value and lambda value. Lambda is the ratio of the characteristic path length between real and 100 random networks, which quantifies the overall routing efficiency of a network (Zhang et al., 2019). The lambda values of SIVD and AD patients were obviously increased in the present study. It was suggested that the network integration in SIVD and AD patients was disrupted. E_g_ and E_loc_ respectively measures the ability of parallel information transmission over the network and the fault tolerance of the network (Zhang et al., 2019). The E_g_ and E_loc_in SIVD patients and E_g_ in AD patients were significantly reduced compared with NC, which indicated that global integration of the structural network was disrupted and information processing was impaired in the AD and SIVD groups (Bai et al., 2012). Lp, a measurement of the average of the nodal shortest path length, reflects the speed of information transfer to the whole brain (Li et al., 2017). Significantly increased Lp in SIVD indicated the disrupted global integration of the structural network. When SIVD patients compared with AD patients, SIVD patients performed lower E_g_, E_loc_ and higher Lp, lambda, suggesting that the destruction of white matter structure in the SIVD group was worse than that in the AD group.

BC is the fraction of all shortest paths in the network that pass through a given node (Fortanier et al., 2019). Decreased nodal BC means reduced nodal importance in the network. Compared with NC, AD patients demonstrated significantly reduced BC in the left precuneus. A previous study found that inappropriate atrophy existed in the precuneus in AD patients and the precuneus played an important role in the default mode network (Abu-Akel and Shamay-Tsoory, 2011; Hojjati et al., 2018). Besides the left precuneus, AD patients showed decreased BC in parietal and prefrontal cortices, such as the right postcentral, left superior parietal gyrus, right rolandic operculum, left inferior frontal gyrus (orbital part), and left superior frontal gyrus (medial part). Some studies suggested that atrophy of the AD brain may initiate in the inferior parietal cortices and spread to the prefrontal cortices (McDonald et al., 2009). These structural alterations may be linked to the corresponding BC changes in these regions and illuminate one of the underlying reasons of cognitive dysfunction in AD patients.

Executive dysfunction is the main clinical symptom of SIVD patients which may be correlated with the integrity of prefrontal-subcortical circuits (Román et al., 2002). Compared with NC, the BC of the left anterior cingulate gyrus in SIVD patients was significantly decreased in the present study. The cingulum, a complex structure that interconnects the frontal, parietal, and medial temporal regions (Bubb et al., 2018), plays an important role in executive function and episodic memory. The impairment of the cingulum may result in an executive dysfunction and memory deficit in SIVD patients. Additionally, the BC was decreased in some prefrontal cortices such as the right superior frontal gyrus, right superior frontal gyrus (orbital part), and right inferior frontal gyrus (opercular part), as well as in the right putamen and right superior occipital gyrus in SIVD patients. Some researchers suggested that the volume of the putamen was smaller and that the occipital gyrus was thinner in SIVD patients (Seo et al., 2010; Thong et al., 2014). The occipital gyrus is the main origin and destination of long-association fibers, among which the inferior fronto-occipital fasciculus plays an important role in attention and visual processing. Therefore, alterations in the structural occipital gyrus may lead to function deficits in attention and visual processing (Catani and Thiebaut De Schotten, 2008).

There were some differing main brain regions involved in the structural network in AD and SIVD patients. It was suggested that the left precuneus was vulnerable to damage in AD patients while the right putamen was vulnerable to damage in SIVD patients in the present study. The prefrontal and frontal cortices and subcortical regions were the main regions affected in the SIVD group which was consistent with a previous study (Yi et al., 2012; Thong et al., 2014). As to the diagnostic efficiency of nodal BC in differentiating SIVD and AD patients, the AUC of the right putamen was 0.946 which was the highest. Therefore, the change of BC in the right putamen could identify and distinguish SIVD patients and AD patients.

Structural connections in frontal-prefrontal regions, frontal-subcortical regions, and prefrontal-subcortical regions were reduced in SIVD patients in the present study. Previous studies demonstrated decreased frontal-subcortical connections in SIVD patients (Sang et al., 2020), which was in line with the present study. Besides, the structural connections between prefrontal and frontal regions were reduced which supposedly resulted in their damage in SIVD patients. AD patients mostly manifested decreased structural connections in the temporal and occipital regions and increased connections in the frontal and prefrontal regions in the present study. In the study of McDonald et al. (2009), AD patients with mild cognitive impairment suffered from atrophy of the temporal and occipital cortices. Structural alterations in the temporal and occipital cortices may explain the decreased structural connections in these regions which are related to function deficits. However, increased structural connections in the frontal and prefrontal regions could potentially compensate for function deficits in AD patients.

As discussed above, although most of the conclusions from the present study were similar with those from previous studies, there were still many new findings. First, the structure of the precuneus was deconstructed in AD patients, which illuminated part of underlying reasons for cognitive dysfunction, while AD patients had increased prefrontal-frontal structural connections, which were supposed to compensate for function deficits. Second, the structure of occipital cortices were impaired in SIVD patients, which pertained to the function deficits of attention and visual processes. Finally, the BC change of the right putamen had the highest AUC in the ROC analysis, suggesting that it was suitable to differentiate between SIVD patients and AD patients.

There were several limitations in the present study. First, the sample size of the SIVD group was quite small, which needs to be enlarged in future studies. Second, the intra-individual and intra-group differences in global cognition were not considered sufficiently. Although there were no statistical differences in MMSE and MoCA scores between AD and SIVD patients, and there was no statistical difference in the distribution of severity of cognitive dysfunction between AD and SIVD patients, the confounding factor of the severity of cognitive impairment existed. AD and SIVD patients need to be further subdivided in a future study. Third, the AD patients were recruited by clinical criteria which had not been proved by pathology.

## Conclusion

White matter structural network analysis including the topological changes of the network, especially the BC change in the right putamen may be a potential and promising method for differentiating AD and SIVD patients.

## Data Availability Statement

The raw data supporting the conclusions of this article will be made available by the authors, without undue reservation.

## Ethics Statement

The studies involving human participants were reviewed and approved by the ethics committees of the First Affiliated Hospital of Soochow University. The patients/participants provided their written informed consent to participate in this study.

## Author Contributions

HD and YW designed this study. MF, YL, ZW, ZS, and MM collected the patients’ data. The cognitive functions of all the subjects were evaluated by YZ. Each author participated in writing the article. Each author gave final agreement to be accountable for all aspects of the work in ensuring that questions related to the accuracy or integrity of any part of the work are appropriately investigated and resolved. All authors contributed to the article and approved the submitted version.

## Conflict of Interest

The authors declare that the research was conducted in the absence of any commercial or financial relationships that could be construed as a potential conflict of interest.
